# Different intestinal ecological niches drive the divergent evolution of probiotics in the gut

**DOI:** 10.1093/ismeco/ycaf023

**Published:** 2025-02-06

**Authors:** Zhe Han, Denggao Huang, Xinlei Liu, Wenyao Ma, Weipeng Cui, Shuaiming Jiang, Dongyu Zhen, Jiachao Zhang

**Affiliations:** School of Food Science and Engineering, Key Laboratory of Food Nutrition and Functional Food of Hainan Province, Hainan University, Haikou 570228, Hainan, China; Central Laboratory, Haikou Affiliated Hospital of Central South University Xiangya School of Medicine, Haikou 570228, Hainan, China; School of Food Science and Engineering, Key Laboratory of Food Nutrition and Functional Food of Hainan Province, Hainan University, Haikou 570228, Hainan, China; School of Food Science and Engineering, Key Laboratory of Food Nutrition and Functional Food of Hainan Province, Hainan University, Haikou 570228, Hainan, China; School of Food Science and Engineering, Key Laboratory of Food Nutrition and Functional Food of Hainan Province, Hainan University, Haikou 570228, Hainan, China; School of Food Science and Engineering, Key Laboratory of Food Nutrition and Functional Food of Hainan Province, Hainan University, Haikou 570228, Hainan, China; School of Food Science and Engineering, Key Laboratory of Food Nutrition and Functional Food of Hainan Province, Hainan University, Haikou 570228, Hainan, China; School of Food Science and Engineering, Key Laboratory of Food Nutrition and Functional Food of Hainan Province, Hainan University, Haikou 570228, Hainan, China; Collaborative Innovation Center of One Health, Hainan University, Haikou, Hainan 570228, China

**Keywords:** probiotics, divergent evolution, *Lactiplantibacillus plantarum*, intestinal ecological niches

## Abstract

Previously, we described the divergent evolution of probiotics in the gut, which potentially compromises their health-promoting effects. Here, we employed a spatiotemporal multiomic approach to explore the distribution and evolutionary trends of the probiotic *Lactiplantibacillus plantarum* HNU082 (Lp082) in specific-pathogen-free and monocolonized mouse models. Initially, after establishing the inherent differences in the gut microbiota between the small and large intestines, we observed that the small intestine served as the main site for Lp082 survival and colonization. Subsequently, we discovered that the small intestine was the sole site where Lp082 exhibited divergent evolution. Moreover, our research indicated that Lp082 had a more substantial impact on the small-intestinal microbiota than on the large-intestinal microbiota. Consequently, we observed a significantly greater number of closely associated species coevolving with Lp082 in the small intestine than in the large intestine. This suggests that Lp082 faced higher selective pressures within the small intestine, potentially leading to the emergence of a greater number of mutants. Our findings will contribute to the differentiated application of probiotics, enhancing their beneficial effects, and offer insights into the targeted domestication of probiotic strains.

## Introduction

Intestinal microorganisms play a crucial role in maintaining human health and ecological balance [1], with significant differences between the microbial compositions of the small and large intestines [2]. The small intestine, primarily responsible for digestion and absorption, harbors a relatively limited microbial population, predominantly composed of species capable of surviving harsh conditions such as stomach acid and bile [3,4]. In contrast, the large intestine hosts a diverse array of microorganisms that perform various ecological functions, including the breakdown of food residues [5], vitamin synthesis [6], pathogen defense [5], and interaction with the immune system [7]—all of which have profound impacts on host health. Probiotics exert their beneficial effects through colonization in specific regions of the gut. For instance, *Lactobacillaceae* species primarily colonize the distal small intestine [8], where they ferment simple carbohydrates to enhance nutrient absorption, while *Bifidobacterium* species reside in the large intestine [3,8], metabolizing complex carbohydrates to produce short-chain fatty acids that regulate immune responses [9]. *Akkermansia muciniphila*, also predominantly found in the large intestine, degrades mucin and oligosaccharides, producing short-chain fatty acids and propionate [10,11], which contribute to weight control and slow the progression of diabetes [12,13]. Therefore, gaining a precise understanding of the specific gut ecological niches where exogenous probiotics thrive and deliver their benefits is a fundamental cornerstone of probiotics-related research.

In previous studies, we focused on the candidate probiotic Lp082 and used three typical biological models (human, mouse, and zebrafish) to elucidate the phenomenon of adaptive evolution of probiotics in the gut [14]. In another study, by simultaneously gavaging specific-pathogen-free (SPF) mice and germ-free (GF) mice with equal amounts of *Lactiplantibacillus plantarum* HNU082 (Lp082) and *Bifidobacterium animalis* subsp. *lactis* V9 (BV9), we found that the interplay of the gut microbiota was the primary driving force behind the adaptive mutations of probiotics. Most importantly, our research revealed that when distinguishing mutation sites were differentiated by quantity, both Lp082 and BV9 differentiated into two distinct clades in the SPF mouse gut, leading to divergent evolution [13]. Divergent evolution refers to the process by which different species or populations undergo adaptive differentiation within the same ecological niche, gradually evolving traits with functional or morphological differences through selective pressures imposed by varying environmental conditions [15]. These intriguing findings led us to ask the following question: what factors and dynamics underlie the divergent evolution of these clades? Based on the principles of biological evolution and the differences in selection pressures between the large intestine and small intestine, we hypothesize that the different environments in which these communities lived can explain this phenomenon.

To address our hypothesis, we conducted the following experiments using the probiotic Lp082 as the subject of our study. We utilized multiomic techniques, such as metagenomics and metabolomics, and a GF mice model system to investigate the reasons behind probiotic diversification in the intestines. Since previous studies have found, Lp082 can survive in the gut for at least 7 days [13]. We administered a one-time gastric gavage of 200 μl of suspension containing 10^10^ cfu/ml probiotic strain to GF and SPF mice. Then, we collected intestinal contents from the large intestine and small intestine at 1, 3, and 7 days after stopping gavage in SPF mice for isolation of the probiotic strains. For GF mice, we collected and cultured bacterial strains from large-intestinal and small-intestinal contents daily to perform genome analysis. Next, we obtained the complete genome sequences of the isolated strains and compared them with the full genome sequence of the original bacterial strain to identify adaptive single-nucleotide variations (SNVs). We then analysed and compared the SNVs detected in different locations in the intestines of GF and SPF mice to elucidate the fundamental reasons behind the divergent evolution of probiotics.

The primary objective of this study was to address two critical scientific questions: (i) where are the primary ecological niches within the intestines where probiotics predominantly survive and exert their beneficial effects? (ii) What are the fundamental reasons behind the divergent evolution of probiotics? The answers to these questions will contribute to the differentiated application of probiotics, enhancing their beneficial effects, and will provide valuable insights for the targeted breeding of probiotics.

## Materials and methods

### Experimental design

**Figure 1:**
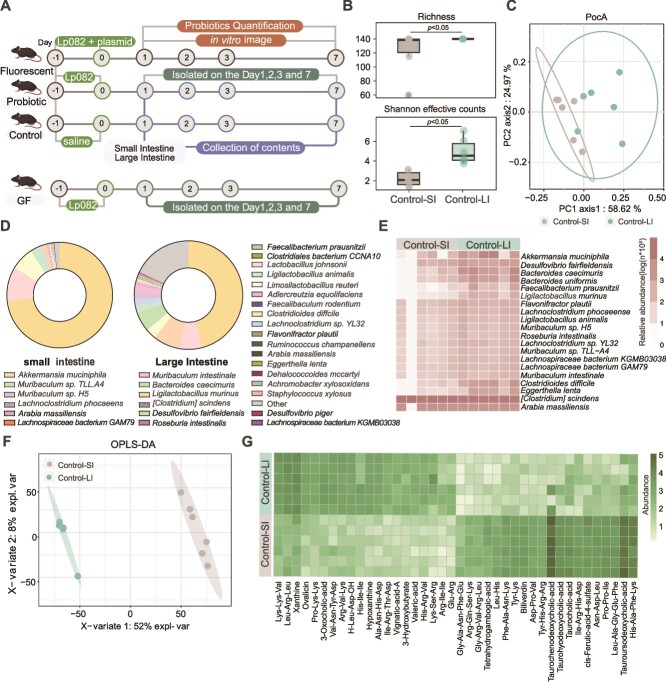
Differences in the microbiome between the large intestine and small intestine. (A) Experimental design. SPF mice were randomly divided into three groups: the probiotic group, fluorescent group, and control group. The probiotic group was orally gavaged with 200 μl of regular Lp082 (10^10^ cfu/ml), the control group received an equivalent volume of saline, and the fluorescent group was orally gavaged with modified Lp082 (containing Lp082 with a fluorescent protein) 200 μl (10^10^ cfu/ml). After one day of gavage, large and small intestinal contents were collected at 1, 3, and 7 days after stopping gavage for metagenomic sequencing, metabolomics analysis, and the isolation and identification of the target bacterial strain. Additionally, the fluorescent group underwent *in vitro* fluorescence imaging and quantitative analysis of Lp082 on days 1, 3, and 7. Moreover, the GF mouse group followed the same gavage strategy as the probiotic group. After stopping gavage, large and small intestinal contents were collected daily for bacterial isolation and identification. (B) Comparison of microbial species diversity within the small and large intestines in the control group. (alpha diversity, richness and Shannon effective counts). (C) Comparison of microbial species structure within the large and small intestines in the control group (based on Bray–Curtis dissimilarity PCoA analysis). (D) In the control group, the species composition of microorganisms within the large and small intestines was assessed at the species level. Different colors indicate distinct species, while the size of the colored blocks reflects their relative proportions within the intestines. (E) Differential microbial species analysis in the control group. Differences in microbial species between the large and small intestine in the control group were assessed using Mann–Whitney analysis, with statistical significance defined as *P* < .05. (F) OPLS-DA analysis between the large and small intestine in the control group. (G) Heatmap showing differential metabolites between the large and small intestine in the control group.

To comprehensively investigate the adaptive evolution of the probiotic Lp082 across different intestinal regions, as well as its co-evolutionary strategies with the host gut microbiota, we designed and conducted a systematic longitudinal study (Fig. 1A). The experiment spanned eight days, beginning with a probiotic intervention on the first day, followed by seven days of monitoring the dynamic interactions between the probiotics and the host gut microbiota. Forty-eight SPF C57 mice were randomly divided into two groups: a probiotic group (24 mice) and a control group (24 mice), with each mouse housed individually. All procedures involving experimental animals were conducted in accordance with protocols approved by the Animal Research Committee of Hainan University and compliedwith the Guide for the Care and Use of Laboratory Animals (Ethics No. HNUAUCC-2023-00174). On day one (day 0 of sample collection), mice in the probiotic group were administered 200 μl of a 10^10^ cfu suspension of Lp082, while control group mice received an equivalent volume of saline. On days 1, 3, and 7 post-gavage, six mice from each group were euthanized at random. Subsequently, the entire gut was dissected into two segments—the small intestine (duodenum, jejunum, ileum) and the large intestine (cecum, colon, rectum)—and the contents of both the small and large intestines were collected, yielding 72 samples (6 mice * 2 groups * 2 sections * 3 time points) for subsequent metagenomic and metabolomic analysis.

To further investigate the evolutionary dynamics of the Lp082 strain within the gut environment, we isolated and cultured Lp082 from the collected small and large intestinal contents on days 1, 3, and 7, followed by identification using specific primers. Whole-genome sequencing was then employed to systematically analyze the genetic variations of the strain during its evolution in the gut. Additionally, we utilized a GF mouse model, introducing Lp082 via the same gavage procedure. During the experiment, three mice were randomly euthanized daily, and their small and large intestinal contents were collected for Lp082 isolation and culturing, followed by whole-genome sequencing to examine the genetic variations under conditions lacking selective pressure from the gut microbiota.

Furthermore, to accurately describe the distribution of Lp082 within the murine gut, 9 SPF C57 mice were gavaged on day 0 with 200 μl of a modified Lp082 bacterial suspension (expressing red fluorescent protein) at a concentration of 10^10^ cfu/ml. On days 1, 3, and 7 post-gavage, three mice were randomly selected for *in vitro* imaging to observe the distribution of Lp082. After imaging, the contents of the small and large intestines were collected, and real-time quantitative polymerase chain reaction (qPCR) was performed to quantify the abundance of Lp082 across different intestinal segments.

### Fecal shotgun metagenomic sequencing and analysis

To investigate the dynamics of gut microbiota across different intestinal regions following exogenous probiotic intervention, we performed shotgun metagenomic sequencing on small and large intestinal content samples from probiotic and control groups on days 1, 3, and 7. Deoxyribonucleic acid (DNA) was isolated and purified using the QIAamp DNA Stool Mini Kit, with quality assessed by 0.8% agarose gel electrophoresis, purity measured by Nanodrop (OD260/280), and concentrations quantified using Qubit 2.0. Sequencing was conducted by Beijing Novogene Technology Co., Ltd. on the Illumina HiSeq 2500 platform, generating paired-end libraries (2 × 150 bp). Raw data underwent quality control via fastp and host genome alignment to remove host-derived DNA fragments. Taxonomic and functional profiling were carried out using MetaPhlAn3 for microbial species identification and relative abundance estimation [16], and for functional profiling based on the UniRef90 database [17].

### Untargeted Metabolomic profiling and analysis

Non-targeted metabolome analysis of the large and small intestine contents of the probiotic group was performed by Wuhan Metawell Biotechnology Co., Ltd. Detailed experimental methods and analysis techniques are shown in the supplementary materials.

To illustrate the effects of probiotics on the metabolism of different gut microbiota in detail, we performed orthogonal partial least squares discriminant analysis (OPLS-DA) of the metabolomics data using the “mixOmics” package in R, with results visualized in Figs 1F, 4E, and 5C. Differential analysis was performed using the online platform Metware Cloud (https://cloud.metware.cn), and results were visualized using the “pheatmap” package.

### *Lactiplantibacillus plantarum* HNU082 fluorescent labeling and *in vitro* fluorescence imaging

To precisely determine the distribution of the probiotic Lp082 in the mouse gut, we introduced the pMG36e-GFP plasmid into the Lp082 strain, enabling stable expression of red fluorescent protein. Detailed methods are provided in the Supplementary Materials. For accurate visualization of Lp082, we utilized an *in vitro* multimodal imaging system (IVIS Spectrum, PerkinElmer) to capture the fluorescence signal emitted by the modified strain. Mice were euthanized prior to imaging to improve clarity, and their entire intestinal tracts were dissected and rinsed with 0.85% physiological saline to remove residual fluid and blood. The intestines were then spread on a clean culture dish for fluorescence imaging. To validate the specificity of the fluorescent signal, comparative imaging was also performed on control mice gavaged with the original Lp082 strain.

### Quantification of Lactiplantibacillus plantarum HNU082 in intestinal content samples

To accurately quantify the presence of Lp082 in different sections of the mouse intestines, we first standardized the weight of the collected contents from both the large and small intestines and extracted DNA using the FastPure Bacteria DNA Isolation Mini Kit. The concentration of a previously prepared Lp082 standard DNA was determined using a microplate spectrophotometer to calculate the copy number for constructing standard curves. In the subsequent qPCR experiment, conducted with an Analytik Jena fluorescence quantitative gene amplification instrument, the Ct values were used to perform linear regression against the logarithm of the template copy number, enabling the calculation of Lp082 copy numbers in various intestinal segments. Further details on the reaction system, procedures, and primers are provided in the Supplementary materials.

### Isolation of strains from large and small intestine contents

To select for Lp082 from the complex content samples, we followed the experimental procedure described in Huang *et al.*’s study [14]. We diluted the large and small intestine contents, collected in the aforementioned experiments, with 0.85% sterile saline. Subsequently, we evenly spread these diluted samples on selective culture plates containing antibiotics. After 48 hours of incubation at 37°C, colonies were picked and subjected to PCR verification using specific primers for Lp082. Confirmed Lp082 isolates were stored at −80°C and later used for whole-genome resequencing.

### Whole-genome resequencing of isolated strains

For whole-genome resequencing, DNA was extracted from the isolated strains. High-throughput sequencing was performed on the Illumina HiSeq 2500 platform, generating paired-end reads (2 × 150 bp) libraries for each bacterial sample. Quality control was carried out using FastQC, and AdapterRemoval (v2.1.7) was performed to eliminate any adapter contamination [18]. Subsequently, quality correction based on K-mer frequencies was conducted using SOAPec (v2.0) software.

### Single-nucleotide variations calling and construction of phylogenetic tree

To obtain genetic variation information for Lp082 isolates from the small intestine, large intestine, and GF mice, FASTQ files were aligned to the reference genome using Bowtie2 [14,19], generating SAM files. These were converted to BAM format, sorted, and indexed with Samtools [20], followed by variant calling with Bcftools to identify SNV sites. The number of SNVs in isolates from different regions was visualized using GraphPad Prism (Version 9.5.1), while SNV density plots were generated with the stringr, circlize, and grid packages in R. Gene mutation frequency was displayed using the “pheatmap” package in R. The SNVs were then used to calculate the genetic differences between each isolate and the reference strain, and a neighbor-joining tree was constructed with MEGA-X to reflect evolutionary relationships, which was further refined using the online tool iTOL and the “itol.toolkit” package in R [21,22].

### Construction of co-occurrence networks and annotation of single-nucleotide variations associated with correlated species

To investigate potential interactions between Lp082 and the host gut microbiota across different intestinal segments, we aggregated metagenomic data from multiple time points for the SPF-probiotic-SI and LI groups and constructed co-occurrence networks for both the large and small intestines using the WGCNA package in R and Spearman’s correlation (R > 0.4, *P* < .05), with visualization in Cytoscape 3.4.0. Additionally, to examine co-evolutionary dynamics between the gut microbiota and Lp082 following intervention, we employed inStrain to identify SNVs in metagenomic data from associated strains. Reference genomes were obtained from NCBI, and libraries were constructed using Bowtie2. FASTQ files were aligned to the reference genomes to generate SAM files, which were converted to sorted and indexed BAM files using Samtools. SNVs were identified using inStrain (v1.0.0) and visualized with the pheatmap package [23].

### Statistical analysis

All schematic figures and workflows were created using BioRender (https://app.biorender.com/). To describe gut microbiota dynamics across intestinal segments post-probiotic intervention, we performed alpha diversity analysis (Shannon effective counts and Richness) and principal coordinate analysis (PCoA) on taxonomic profiles from metagenomic data using the “vegan” package in R. Box and scatter plots were visualized with “ggplot2”. The Kruskal–Wallis test identified species differences between groups, visualized with “ggplot2” and “pheatmap”. Differential pathways were analyzed with “DESeq2” and visualized with “ggplot2”. The species composition of the microbiota in the large and small intestines of the control group was displayed using GraphPad Prism (Version 9.5.1).

## Results

### The microbiomes of the mouse small intestine and large intestine showed significant differences

Here, by performing metagenomic and metabolomics, we found that the α-diversity of the small intestine was consistently significantly lower than that of the large intestine (Richness and Shannon effective counts, Mann–Whitney, *P* < .05; Fig. 1B), which was likely associated with the difference in pH between the small intestine and the large intestine [24]. The PCoA based on Bray–Curtis dissimilarities revealed a distinct separation in the microbial composition between the small intestine and large intestine (Fig. 1C). At the species level, while *Ligilactobacillus murinus* was a major component in both the small intestine and large intestine, its proportion was notably higher in the small intestine (Fig. 1D). Moreover, we identified 102 differentially abundant species between the small intestine and large intestine (only the top 20 most abundant taxa are shown; Mann–Whitney, *P* < .05). *A. muciniphila* and *Faecalibacterium prausnitzii* had significantly higher relative abundances in the large intestine than in the small intestine (Fig. 1E). Furthermore, the significant differences in the metabolic profiles between the small intestine and large intestine was descripted (Fig. 1F). Specifically, when we focused on differentially abundant metabolites between the large-intestinal and small-intestinal microbiomes, we found that the levels of metabolites such as Xanthine, Hypoxanthine, and Leu-Arg-Leu were significantly higher in the large intestine, whereas metabolites, such as Taurochenodeoxycholic-acid and Taurohyodeoxycholic-acid, were more abundant in the small intestine (Fig. 1G).

### The small intestine was the primary site for *Lactiplantibacillus plantarum* HNU082 survival, colonization, and divergent evolution

**Figure 2:**
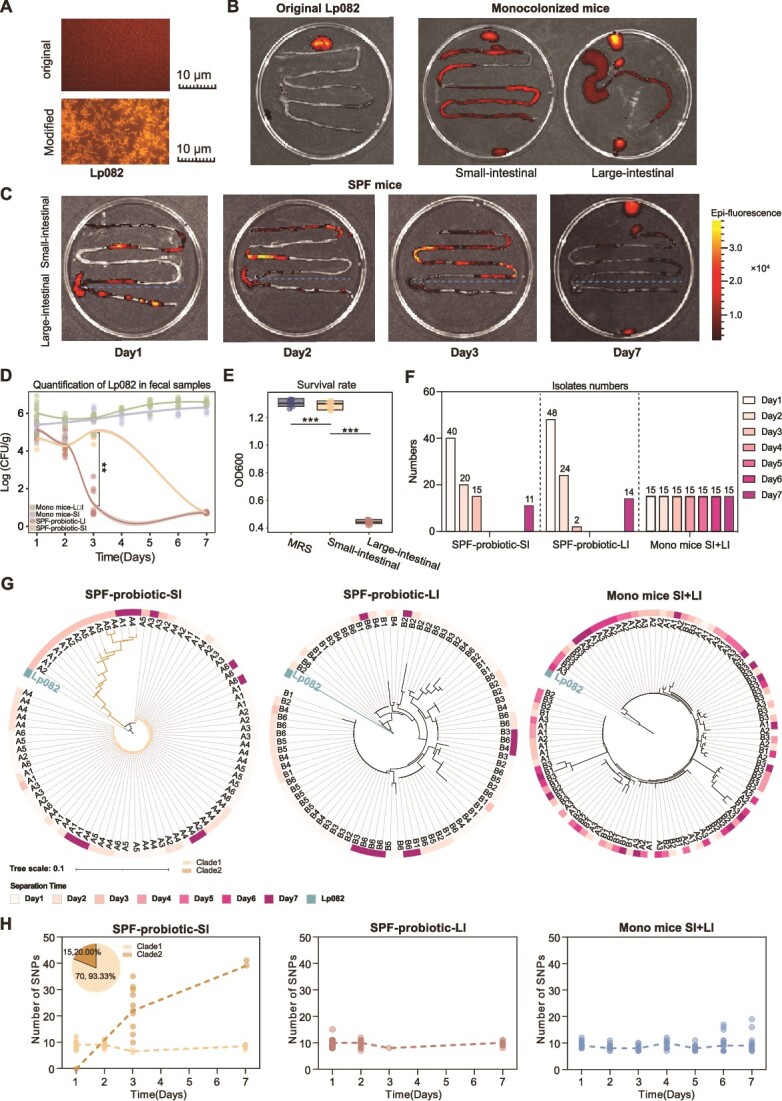
Distribution and evolution of probiotics in the intestine. (A) Luminescence of transduced strains compared to the original strains under an upright fluorescence microscope. (B and C) *In vitro* imaging photos based on IVIS, with colors ranging from red to yellow. Closer to yellow indicates higher abundance of the strain at that location. Using the blue dashed line as a boundary, the area above the blue dashed line represents the small intestine, while the area below corresponds to the large intestine. (D) Quantitative results of Lp082 in the large and small intestine content at different time points in the probiotic and mono groups. The bottom part shows the number of isolated strains of Lp082 at different time points from the large and small intestine content in both probiotic and mono mice. (E) The OD600 of Lp082 after 12 hours of culture in standard MRS, large intestinal simulation medium, and small intestinal simulation medium. (F) The number of Lp082 isolates in the SPF and mono mice groups. (G) Phylogenetic tree constructed based on SNVs of Lp082 isolates from the SPF and mono groups. (H) The number of annotated SNVs in isolates obtained from the large and small intestine of the SPF group and the mono group at each time point.

To precisely identify the survival and colonization sites of Lp082 in the intestinal tract, we employed a fluorescence labeling strategy to genetically modify the Lp082 (Methods). We observed that the modified Lp082 exhibited strong fluorescence signals under a fluorescence microscope, indicating stable expression of the fluorescent protein (Fig. 2A). Through gavage and *in vitro* imaging of SPF and Mono mice, we observed that, the strain rapidly occupied the primary ecological niches within the gut of Mono mice, exhibiting widespread distribution across the entire intestinal tract (Fig. 2B). In contrast, in SPF mice, the strain predominantly survived and colonized the small intestine. However, over time, especially within 7 days after gavage cessation, the imaging results showed that only a minimal number of surviving strains remained in the small intestine (Fig. 2C).

Furthermore, we quantitatively assessed the strain in the intestinal contents from different segments at various time points post-intervention using strain-specific primers (Methods, Fig. 2D). The quantitative results were highly consistent with the imaging observations, indicating that the cells numbers of this strain were significantly higher in the small intestine than in the large intestine (SPF-probiotic-LI VS SPF-probiotic-LI, *P* < .05). Studies based on the Mono mice model demonstrates that there was no significant difference in the survival and colonization of the probiotic strain between the small and large intestines post-ingestion. After a single gavage, the strain could rapidly and stably occupy the entire intestinal ecological niche in the absence of additional microbial selection pressure. These findings further suggest that host factors exert a much smaller influence on the colonization of probiotic strains compared to microbial factors.

Moreover, we measured the pH and total bile acid concentrations in the small and large intestines of SPF mice, and cultured the original Lp082 strain in media designed to mimic the intestinal environment, using hydrochloric acid and bovine bile salts (large intestine: pH = 6, total bile acid concentration = 735.21 μmol/L; small intestine: pH = 7, total bile acid concentration = 486.12 μmol/L; Fig. S1B and C). After 12 hours of incubation with the same inoculum, the growth rate of Lp082 in the small intestine-mimicking medium was significantly higher than that in the large intestine-mimicking medium (Fig. 2E). These results collectively demonstrate that in the SPF mice, the small intestine was the primary spatial location for Lp082 survival and colonization, with some strains exhibiting limited survival and replication upon reaching the large intestine, eventually being rapidly eliminated from the body.

After addressing the issue of Lp082 survival and colonization sites from an ecological perspective, we delved into the question of strain evolution within the intestinal tract. By utilizing selective culture media and strain-specific primers, we isolated 85 Lp082 mutant strains from the small intestine of SPF mice, 86 Lp082 mutant strains from the large intestine, and 105 Lp082 mutant strains from GF mice. We performed whole-genome resequencing of these isolates and annotated the mutation sites (Methods, Fig. 2F). Based on the annotated mutation sites, we classified the isolated strains according to different intestinal segments and different mouse models to construct a phylogenetic tree (Fig. 2G). Interestingly, the strains isolated from the small intestine of SPF mice exhibited divergent evolution over time. One branch, encompassing 70 strains, exhibited only approximately 10 mutations, indicating a relatively stable genome throughout the colonization phase. In contrast, another branch containing 15 strains underwent a substantial number of mutations, with the highest number of mutation sites reaching 40. The key turning point for probiotic strain evolution was found to be the third day post-ingestion, as all strains with numerous mutation sites began divergent evolution from the third day onwards, ultimately reaching 18.8% of the total isolated strains in that intestinal segment by the seventh day (Fig. 2H). However, no evidence of divergent evolution was observed in the entire large intestine or in the Mono mice model, where all isolated strains carried approximately 10 mutation sites and closely resembled the “weak mutation” strains of the first evolutionary branch of the Lp082 strains isolated from the small intestine of SPF mice. The above results clearly show that divergent mutations in Lp082 only occurred in the small intestine of SPF mice, and the third day after ingestion of this strain was a critical time point for evolution.

### Genomic characteristics of high-mutational branches in divergent evolution

**Figure 3:**
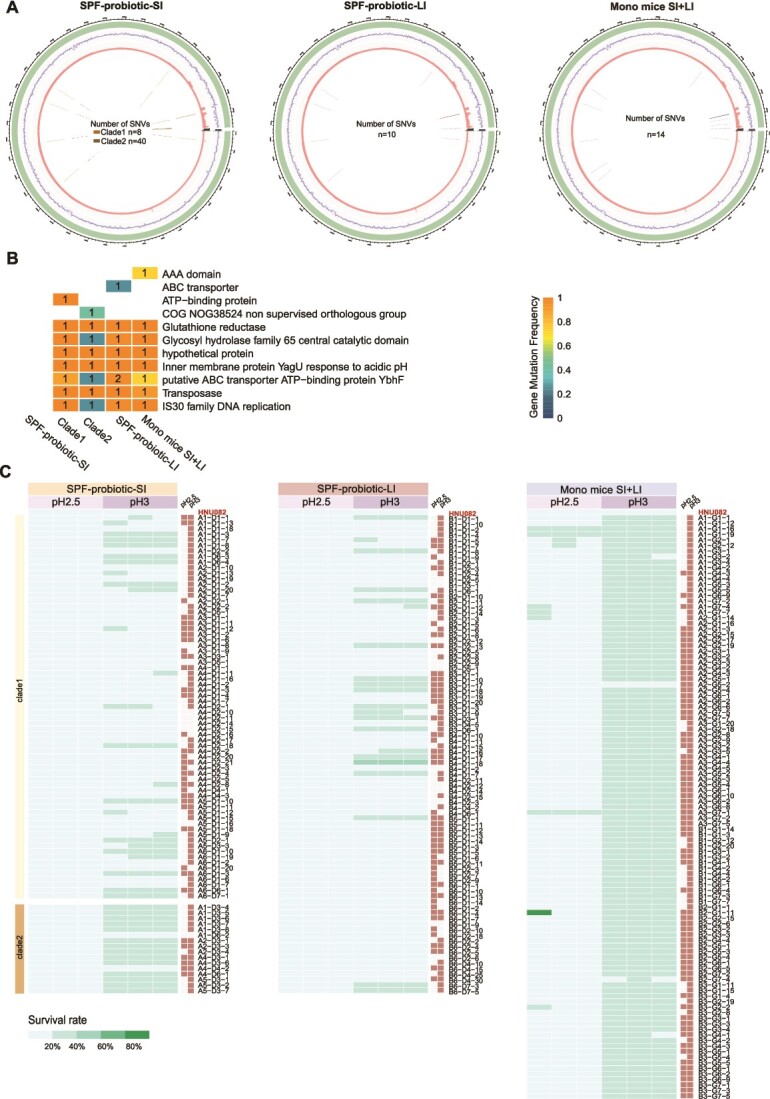
**Functional annotation of mutated genes in Lp082 at different sites.** A. Distribution of SNVs in Lp082 isolates from different groups. B. A total of 11 genes had mutations in all isolates, with the intensity of the blocks representing the frequency of mutations. The numbers on the color blocks represent the median number of SNVs in each group of isolates. C. The acid resistance assay of mutant strains and the original strain from the SPF and mono groups, with greater intensity indicating higher survival rate. Solid symbols denote significant difference from the original Lp082, while open symbols indicate no significant difference.

Following the identification of the evolutionary dynamics of the Lp082 within the gut, we further investigated the differences in the distribution of SNVs across the genome among Lp082 isolates from the small intestine, large intestine, and Mono mice (Methods, Fig. 3A). Therefore, we compiled the SNVs from the isolates of the three groups and constructed Circos plots to visualize the genomic distribution of SNVs across the three groups. The results indicated that the genomic distribution of SNVs in Lp082 isolates from the large intestine and small intestine (clade 1) was similar, while SNVs in clade 2 isolates from the small intestine were predominantly concentrated at the terminal regions of the genome (SNVs: small-intestine clade 1, *n* = 8; clade 2, *n* = 40, large-intestine, *n* = 10). In Lp082 isolates from GF mice, the genomic distribution of SNVs was more widespread (SNVs: GF, *n* = 14).

**Figure 4:**
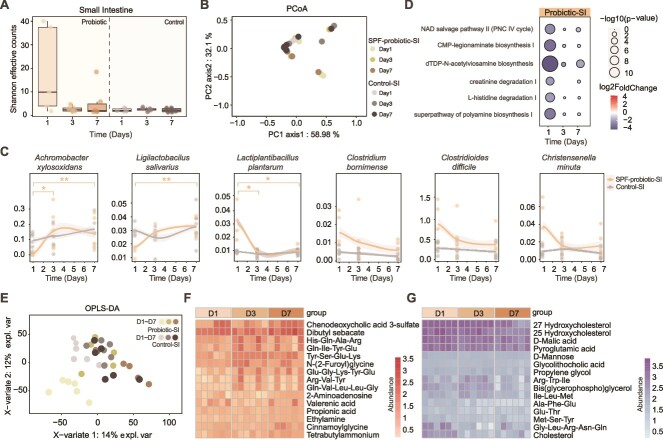
Response of the small intestinal microbiota to probiotic intervention. (A) Comparison of species diversity (alpha diversity, Shannon effective counts) between the probiotic group and the control group based on small intestinal microbiota. (B) Comparison of microbial community structure in the small intestine between the probiotic group and the control group at different time points (based on Bray–Curtis dissimilarity PCoA analysis). (C) Species with significantly altered abundances in the probiotic group compared to the control group after probiotic intervention, based on the Mann–Whitney test. Asterisks indicate statistical significance (*P < .05, **P < .01, ***P < .001). (D) Heatmap showing the sustained changes in metabolite levels that were significantly altered compared to the control group. On the left are metabolites with continuously upregulated expression. On the right are metabolites with continuously downregulated expression. (E) OPLS-DA analysis between the probiotic group and the control group’s small intestine at different time points. (F and G) Metabolic pathways significantly different between the probiotic group and the control group compared.

**Figure 5:**
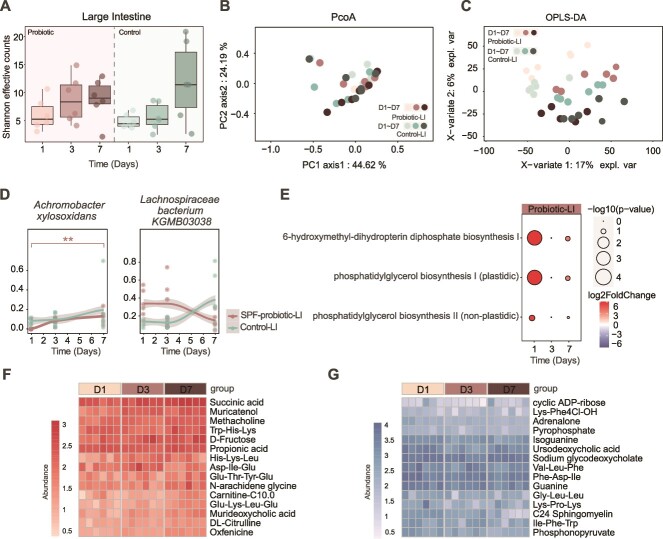
Response of the large intestine microbiota to probiotic intervention. (A) Comparison of species diversity (alpha diversity, Shannon effective counts) in the large intestine microbiota between the probiotic group and the control group. (B) Comparison of microbial community structures in the large intestine between the probiotic and control groups at different time points based on Bray–Curtis dissimilarity PCoA analysis. (C) OPLS-DA analysis between the probiotic group and the control group in the large intestine at different time points. (D) Species with significantly different abundances in the probiotic group compared to the control group after probiotic intervention, based on the Mann–Whitney test. Asterisks indicate statistical significance (*P < .05, **P < .01, ***P < .001). (E) Significantly different metabolic pathways between the probiotic group and the control group. F. Heatmap displaying significantly upregulated metabolites in the probiotic group compared to the control group. (G) Heatmap displaying significantly downregulated metabolites in the probiotic group compared to the control group.

Furthermore, we evaluated the potential impact of these mutation sites on gene function. Genes encoding AAA domain and DNA helicase were mutated in all three groups; these genes are primarily involved in functions such as catalyzing DNA synthesis (Fig. 3B). Notably, the protein YagU stands out as a gene exhibiting high-frequency mutations within the branch characterized by high mutational rates in the context of divergent evolution. The primary function of this gene is associated with acid resistance. Subsequent studies have indicated that YagU serves as an acid stress response gene, with its expression contributing to the enhancement of bacterial acid resistance, thereby enabling adaptation to the external environment [25]. To investigate the function of this gene, we conducted acid tolerance experiments on both the original strain and all mutant strains (Fig. 3C). The results showed that under pH 2.5 conditions, the survival rate of the 152 strain mutants was significantly higher than that of the original strain (large intestine: 47, small intestine clade1: 29, clade2: 6, Mono mice:70). Furthermore, under pH 3 conditions, the survival rate of the 235 strain mutants was also significantly higher than that of the original strain (large intestine: 61, small intestine clade1: 54, clade2: 15, Mono mice: 105). The strains adapted through intestinal evolution exhibited significantly improved acid tolerance, suggesting that the YagU may promote the survival and colonization of probiotics in different intestinal environments by enhancing acid tolerance.

### Differential responses of the small-intestinal and large-intestinal microbiotas to probiotic intervention

The interaction with the host’s native gut microbiota forms the foundation for the colonization and efficacy of probiotics within the gut [26,27]. At the single-strain level, we have clearly elucidated the colonization and evolution strategies of the probiotic. However, the response of the gut microbiota in different intestinal segments to the introduction of this strain remains unexplored. By employing metagenomic and metabolomic techniques, we tracked dynamic changes in the gut microbiota across various regions of the mouse intestine to investigate their responses to probiotic intervention.

We first focused on the response of the small intestinal microbiota to probiotic intervention. In terms of α-diversity, microbial diversity initially increased following probiotic administration but then decreased, indicating that the introduction of the probiotic rapidly enhanced microbial diversity in the small intestine. After a sharp change on day 1, the microbial community gradually stabilized and returned to near-baseline levels (Fig. 4A). PCoA based on Bray–Curtis dissimilarity (β-diversity) revealed structural differences between the probiotic and control groups. Consistent with the α-diversity results, the microbial community structure exhibited significant divergence on day 1 but returned to a near-control state by day 7, indicating a gradual stabilization and convergence of the community structure toward that of the control group (Fig. 4B). We also examined changes in the relative abundance of responsive species following probiotic intervention (Mann–Whitney, *P* < .05; Fig. 4C). After probiotic intake, *Achromobacter xylosoxidans* and *Ligilactobacillus salivarius* showed significant increases in relative abundance, while *Clostridium bornimense*, *Clostridioides difficile*, and *Christensenella minuta* significantly decreased (SPF-probiotic-SI vs. Control-SI, Mann–Whitney, *P* < .05). Regarding differential metabolic pathways, six pathways, including dTDP-N-acetylviosamine biosynthesis and L-histidine degradation I, were significantly downregulated following probiotic intervention (log2fc > 2, adjusted *P* < .05, Fig. 4D). From a metabolic perspective, OPLS-DA shows that on day 1, there is a significant difference in the metabolite composition of intestinal contents between the probiotic-SI group and the control group, with this difference gradually returning to baseline levels at subsequent time points (Fig. 4E). After probiotic intervention, including chenodeoxycholic acid 3-sulfate and Tyr-Ser-Glu-Lys were significantly upregulated, while 281 metabolites such as 27-hydroxycholesterol and pyroglutamic acid were significantly downregulated (log2fc > 2, adjusted *P* < .05, Fig. 4F and G).

Interestingly, due to the distinct colonization sites of the probiotic, no significant changes in α-diversity or microbial community structure were observed in the large intestine following probiotic intervention, with no notable differences between the probiotic and control groups in microbial diversity or community structure (PCoA; Fig. 5A and B). Only *A. xylosoxidans* and *Erysipelotrichaceae KGMB03038* showed significant responses to probiotic intake (Mann–Whitney, *P* < .05), with the former increasing and the latter decreasing in abundance (Fig. 5D). Additionally, three metabolic pathways were significantly upregulated, including 6-hydroxymethyl-dihydropterin diphosphate biosynthesis I and phosphatidylglycerol biosynthesis (log2fc > 2, adjusted *P* < .05; Fig. 5E). While the overall metabolic structure remained unchanged (Fig. 5C), certain metabolites, such as succinate and acetylcholine, were upregulated, whereas 27-hydroxycholesterol and pyroglutamic acid were downregulated (log2fc > 2, adjusted *P* < .05; Fig. 5F and G). Collectively, the small intestine exhibited a more pronounced response to the probiotic, confirming it as the primary niche for Lp082.

### The small-intestinal microbiota drives the divergent evolution of Lactiplantibacillus plantarum HNU082

**Figure 6:**
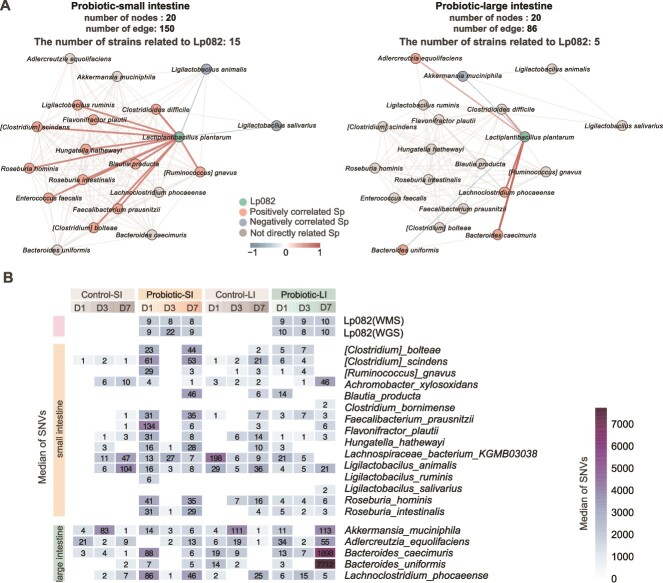
The microbial co-occurrence network reveals the sources of intestinal selection pressure. (A) The microbial co-occurrence network depicts the species closely associated with Lp082 in different gut locations. (B) The heatmap displays the co-evolution of microbial species associated with LP082 in the large intestine and small intestine after probiotic intake. The median number of SNVs in each microbial species is marked on the color block.

Having identified the spatial locations where Lp082 undergoes divergent evolution and the responses of different gut segments to Lp082 intervention, we continued to explore the fundamental question of why Lp082 exhibits divergent evolution. First, we constructed microbial co-occurrence network interaction graphs for the small and large intestines to identify species significantly correlated with Lp082 (Spearman correlation, r > 0.4, *P* < .05; Methods, Fig. 6A). Figure 6A reveals that in the small intestine, there were 15 species directly interacting with Lp082, encompassing both cooperative interactions, represented by *Ligilactobacillus ruminis*, *Flavonifractor plautii*, and *Lachnoclostridium phocaeense*, and competitive interactions, represented by *Ligilacrobacillus animalis* and *Ligilactobacillus salivarius*. Then, we collected reference genomes of all related strains from NCBI and utilized whole metagenome sequencing (WMS) data to obtain genetic variation information of the associated strains in order to investigate their co-evolutionary dynamics (Methods, Fig. 6B). Consequently, these species coevolved with Lp082 following probiotic intake, with *F. plautii* exhibiting as many as 134 mutation sites after probiotic intervention. In the large intestine, only five species, namely, *A. muciniphila*, *Bacteroides uniformis*, *Bacteroides caecimuris*, *Adlercreutzia equolifaciens*, and *Lachnoclostridium phocaeense*, showed correlations with Lp082. Consequently, only a limited number of species underwent mutations.

Therefore, in the small intestine, Lp082 interacts with a more diverse microbiota, leading to higher selective pressure and faster evolution. In the large intestine, where most species showed no significant correlation with Lp082, there is no driving force for evolution, and strains remain relatively stable, ultimately being excreted as transient microbes. In summary, the high selective pressure mediated by the small-intestinal microbiota is the fundamental driver of the divergent evolution of Lp082.

## Discussion

Although our previous studies have revealed the phenomena of adaptive and divergent evolution of probiotics under intestinal selective pressure, the underlying causes driving this divergence and the specific locations where evolution occurs within the gut remain largely unknown [13]. In this study, we employed multi-omics approaches and utilized SPF and GF mouse models to systematically investigate the colonization sites and evolutionary dynamics of the probiotic strain Lp082 in the host gut. We identified the underlying mechanisms responsible for the divergent evolution observed in the small intestine. Based on *in vitro* imaging and quantitative PCR results, we determined that the primary niche of Lp082 within the gut is the small intestine. By analyzing the mutation profiles of isolates from the small intestine, large intestine, and GF mice at different time points, we found that the number of SNVs in Lp082 isolates from the small intestine was significantly higher than that from the large intestine and GF mice. We also observed divergent evolution of probiotics in the small intestine.

Additionally, based on WMS data, we found that the microbial community in the small intestine is more responsive to the intervention of probiotic Lp082 compared to the large intestine, as indicated by a significant increase in the complexity of its microbial symbiotic network. This further suggests that the small intestine is the primary ecological niche for Lp082. The intricate microbial network in the small intestine intensifies the selective pressure on Lp082, thereby accelerating its evolutionary rate and extent, ultimately leading to divergent evolution. This phenomenon was not observed in the large intestine or in GF mice. Additionally, fewer mutations were detected in Lp082 strains isolated from GF mice, indicating that in the absence of intestinal selective pressure, the mutation frequency of the probiotic is reduced. This finding supports the notion that the higher mutation rate of Lp082 is primarily driven by the elevated selective pressure present in the small intestine.

The discovery that high-intensity intestinal selective pressure can lead to divergent evolution in probiotic strains provides new insights into targeted probiotic domestication [28]. For instance, by altering the composition of the gut microbiota, it may be possible to design specific selective pressures within the gut to domesticate probiotics, enhancing their beneficial functions [29]. This approach could be applied in targeted scenarios, such as leveraging probiotics for specific patient populations in order to achieve precision medicine outcomes. The discovery that high-intensity intestinal selective pressure can lead to divergent evolution in probiotic strains provides new insights into targeted probiotic domestication [28]. Previous studies have shown that it is possible to enhance the probiotic efficacy of bacterial species by altering intestinal selection pressure. For example, changes in gut selective pressure, mediated by systematically altering diet and the complexity of the local microbiota, induced mutations in the probiotic strain *Escherichia coli* Nissle (EcN) related to carbohydrate utilization, stress response, and adhesion, thereby conferring competitive adaptability [30].

However, our study still has some limitations. Our data only revealed the correlation between the evolution of Lp082 and its interactions with other microbial members at the genomic level, lacking direct causal evidence. Therefore, further validation is necessary. In future studies, we plan to integrate transcriptomic and metabolomic data to systematically explore the mechanisms underlying probiotic niche selection and investigate the role of associated strains in the adaptive evolution of exogenous probiotics. In addition, we will focus on comparing the differences in intestinal adaptability and colonization ability between the original strain and the mutant strain.

Overall, our research, utilizing multi-omics approaches and GF mouse models, addresses two key questions: (1) Lp082 primarily colonizes the small intestine, where it exerts its probiotic effects; and (2) the high selective pressure mediated by the small intestinal microbiota is the driving force behind the divergent evolution of Lp082. The answers to these questions provide valuable insights for optimizing probiotic application and efficacy, while also offering important theoretical guidance for the targeted domestication of probiotic strains.

## Supplementary Material

Supplementary_Materials_ycaf023

## Data Availability

All the genome resequencing data and metagenomic sequencing data reported in this paper have been deposited in the NCBI database under project numbers PRJNA1033724 and PRJNA1035164, respectively. Sample A1D1 did not upload metagenomic data due to DNA extraction failure.
