# Supplementary material for: Different intestinal ecological niches drive the divergent evolution of probiotics in the gut
Source: ISME Commun. 2025 Feb 6;5(1):ycaf023. doi: 10.1093/ismeco/ycaf023 (PMC11879351; doi:10.1093/ismeco/ycaf023)
Supplement: Supplementary_Materials_ycaf023 [file supplementary_materials_ycaf023.pdf]

Lactiplantibacillus plantarum HNU082 fluorescent labelling

To accurately localize the distribution of the probiotic Lp082 in the mouse gut, we introduced the pMG36e-GFP plasmid (generously provided by Jiangnan University), which enables stable expression of red fluorescent protein, into the Lp082 strain.

First, we cultured *Escherichia coli* DH5α cells containing the pMG36e-GFP plasmid in LB liquid medium for 16 hours. The plasmid DNA was then extracted using the Sanprep column plasmid DNA mini-prep kit, and its concentration and purity were measured using a microvolume spectrophotometer.

Next, we activated the Lp082 by inoculating it into Man, Rogosa, and Sharpe (MRS) liquid medium and cultured it until it reached the exponential phase. The culture was then transferred to solution A (composed of 13.67 g sorbitol and 1.0 g glutamic acid, diluted to 100 mL with MRS liquid medium). The cells were statically cultured at 37°C until the OD600nm reached approximately 0.5. Then, 5% (V/V) of the culture was transferred to 100 mL of fresh solution A and further cultured until the OD600nm again reached 0.5. The culture was then ice-bathed for 10 minutes and centrifuged at 4°C at 4500 rpm for 10 minutes, and the supernatant was discarded.

The cell pellet was washed with an equal volume of solution B (composed of 163 g sucrose and 0.166 g magnesium chloride, diluted to 500 mL with distilled water). After gently resuspending the cells, they were centrifuged again at 4°C at 4500 rpm for 10 minutes, and the supernatant was discarded. This washing and centrifugation process was repeated. The final cell pellet was resuspended in solution B at 1/100 of the original culture volume, and the cell suspension was aliquoted into pre-cooled 1.5 mL microcentrifuge tubes (100 μL per tube) and stored at -80°C to obtain competent Lp082 cells.

Before the electroporation experiment, the plasmid solution, competent Lp082 cells, and electroporation cuvettes were pre-chilled. We mixed 4-6 μL of the pMG36e-GFP plasmid with 100 μL of competent Lp082 cells, gently tapping the tube to ensure uniform mixing. The mixture was then transferred into a pre-chilled 2 mm electroporation cuvette. Electroporation was performed at 2100V, and immediately after the pulse, the cuvette was removed and placed into 1 mL of pre-chilled recovery

medium. The mixture was transferred to a microcentrifuge tube and incubated at 37°C on a shaker at 220 rpm for 2-6 hours.

Finally, the liquid culture was evenly spread on MRS agar plates containing 1500 μg/mL erythromycin and incubated statically at 37°C for 24 hours. During this process, colonies exhibiting erythromycin resistance were observed and recorded. These colonies were selected and further cultured in MRS liquid medium containing the same concentration of erythromycin for subsequent experiments (Fig. 2A, B).

In vitro fluorescence imaging

To accurately visualize the distribution of the probiotic Lp082 in the mouse gut, we employed an *in vitro* multimodal imaging system (IVIS Spectrum, PerkinElmer) to capture the fluorescence signal of Lp082 in the intestines. To obtain clearer images of bacterial distribution, mice were euthanized prior to imaging, and their complete intestinal tracts were dissected. The intestines were then rinsed with 0.85% physiological saline to remove any residual tissue fluid and blood, after which they were spread out on a clean culture dish for fluorescence imaging. Throughout the imaging process, we also conducted comparative observations with control mice that had been gavaged with the original Lp082 strain (Fig. 2C).

Untargeted Metabolomic Profiling

Untargeted metabolomic profiling of both the probiotic and control group's large and small intestine samples was conducted at Wuhan Metware Biotechnology Co., Ltd.

The procedure for processing intestinal content samples is as follows: Firstly, intestinal content samples were removed from a -80°C freezer and thawed on ice. Subsequently, a mixed solution was prepared using methanol and water in a 7:3 (V/M) ratio, and 400 μL of this mixed solution was added to 20 mg of the sample. The sample mixture was vortexed for 3 minutes to ensure thorough mixing, followed by 10 minutes of ultrasonication in an ice bath. After another 1-minute vortex, the mixture was allowed to stand at -20°C for 30 minutes and then centrifuged at 12,000 rpm for 10 minutes at 4°C. The resulting precipitate was discarded, and 200 μL of the supernatant was

retrieved for subsequent LC-MS analysis.

All samples were analyzed using two LC/MS methods. In positive ion mode, the following conditions were employed: a T3 chromatographic column, solvent A as 0.1% formic acid in water, solvent B as 0.1% formic acid in acetonitrile, a flow rate of 0.4 mL/min, and an injection volume of 4 µL. The LC-MS analysis utilized a gradient elution with changes in solvent B concentration over time (0-2 min, 5%; 2-5 min, 20%; 5-6 min, 60%; 6-7.6 min, 99%; 7.6-10 min, 5%). Another set of samples was analyzed in negative ion mode, using the same elution gradient and program as in the positive ion mode.

Data acquisition was performed in information-dependent acquisition (IDA) mode using Analyst TF 1.7.1 software (Sciex, Concord, ON, Canada). The ion source parameters and TOF MS scan parameters were configured according to the Luo's article to ensure high-quality data acquisition and analysis[1].

Quantification of Lp082 in Intestinal Content Samples

The reaction system was 20µL, including 6µL of 2X HQ SYBR PCR Mix (without ROX), 1µL of DNA template, and 2µL of the sample, with sterile ddH2O added to 20µL. The reaction program included an initial denaturation at 95°C for 30s, followed by 40 cycles of denaturation at 95°C for 10s, annealing at 60°C for 30s, and extension at 72°C for 30s.

References

1. Luo, W.*, et al.* Metabolome analysis shows that ultrasound enhances the lethality of chlorine dioxide against Salmonella Typhimurium by disrupting its material and energy metabolism. *Food Res Int* **162**, 112135 (2022).

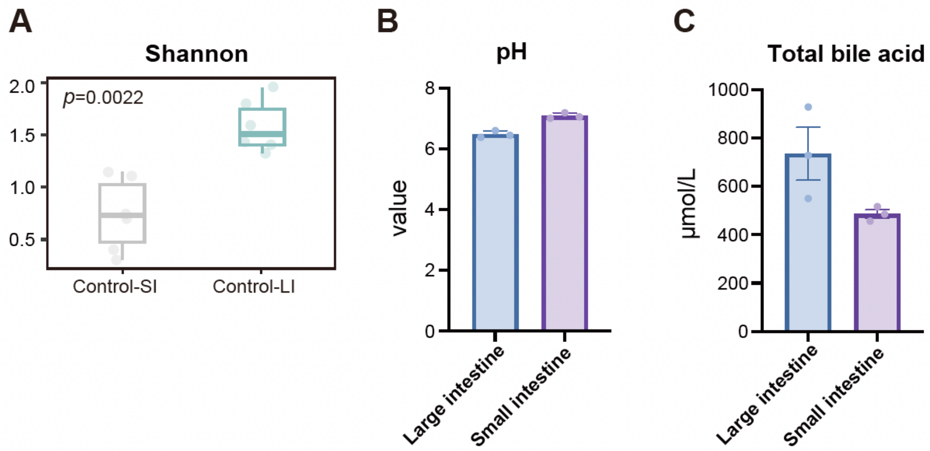

Supplementary Figure1 legend

A. Comparison of microbial species diversity within the small and large intestines in the control group. (Alpha diversity, Shannon)

B. pH values in the large intestine and small intestine. Bars represent the mean pH ± standard error for each group, and individual dots indicate replicate measurements (n = 3).

C. Total bile acid values concentrations (μmol/L) in the large intestine and small intestine. Bars represent the mean ± standard error for each group, and individual dots indicate replicate measurements (n = 3).
